# Type VI Secretion Effectors: Methodologies and Biology

**DOI:** 10.3389/fcimb.2017.00254

**Published:** 2017-06-15

**Authors:** Yun-Wei Lien, Erh-Min Lai

**Affiliations:** ^1^Institute of Plant and Microbial Biology, Academia SinicaTaipei, Taiwan; ^2^Department of Plant Pathology and Microbiology, National Taiwan UniversityTaipei, Taiwan

**Keywords:** type VI secretion system, methodology, effector, toxin-immunity, proteomics, bioinformatics, library, protein-protein interaction

## Abstract

The type VI secretion system (T6SS) is a nanomachine deployed by many Gram-negative bacteria as a weapon against eukaryotic hosts or prokaryotic competitors. It assembles into a bacteriophage tail-like structure that can transport effector proteins into the environment or target cells for competitive survival or pathogenesis. T6SS effectors have been identified by a variety of approaches, including knowledge/hypothesis-dependent and discovery-driven approaches. Here, we review and discuss the methods that have been used to identify T6SS effectors and the biological and biochemical functions of known effectors. On the basis of the nature and transport mechanisms of T6SS effectors, we further propose potential strategies that may be applicable to identify new T6SS effectors.

## Introduction

Cell-to-cell communication and interaction is a central theme for all life forms including single-cell organisms such as bacteria. Gram-negative bacteria have evolved a variety of protein secretion systems to export or import macromolecules across membranes for survival and fitness. The type VI secretion system (T6SS) is a versatile injection machine that can deliver effector molecules into the environment, eukaryotic hosts and prokaryotic competitors. The T6SS mainly functions in a contact-dependent manner to target bacterial competitors for interbacterial competition and eukaryotic hosts for pathogenesis (Durand et al., 2014; Russell et al., 2014a; Cianfanelli et al., 2016b). However, recent reports also suggested that some T6SS effectors may exert their functions extracellularly rather than inside target cells (Wang et al., 2015; Lin et al., 2017; Si et al., 2017).

Typically, the T6SS gene cluster encodes 13–14 conserved core components for machinery assembly and some less conserved accessory proteins and effectors related to T6SS regulation and biological functions (Records, 2011; Basler, 2015; Cianfanelli et al., 2016b). The system consists of a TssJLM (or TssLM) trans-membrane complex (Aschtgen et al., 2008; Ma et al., 2009, 2012; Felisberto-Rodrigues et al., 2011), which serves as a docking site for the TssEFGK baseplate complex (Brunet et al., 2015). TssA, a starfish-like dodecametic complex, connects the TssEFGK baseplate to the Hcp tube and TssBC sheath components for polymerization of this tail structure (Planamente et al., 2016; Zoued et al., 2016). TssB-TssC functions as a contractile sheath, which presumably wraps around the Hcp tube and dynamically propels the Hcp-VgrG puncturing device and associated T6SS effectors across bacterial membranes (Basler et al., 2012). After the firing action, ClpV AAA+ ATPase binds to the contracted TssBC outer sheath for disassembly into subunits, which can be recycled for the next T6SS assembly (Bonemann et al., 2009; Basler and Mekalanos, 2012). Furthermore, Hcp, VgrG, and associated effectors could be translocated into bacterial target cells and reused to assemble new T6SS machineries in target cells (Vettiger and Basler, 2016).

The T6SS has evolved multiple strategies for effector delivery. On the basis of the known effector transport mechanisms, effectors can be classified as “specialized” or “cargo” effectors (Cianfanelli et al., 2016b). Specialized effectors are fused to the C-terminus of T6SS structure proteins, such as Hcp, VgrG, or PAAR (Pro-Ala-Ala-Arg)-domain-containing protein known to sharpen the VgrG spike (Shneider et al., 2013). However, cargo effectors interact directly or require a specific chaperone or adaptor protein for loading onto the lumen of the Hcp tube or VgrG spike. T6SS adaptors/chaperones including DUF4123-, DUF1795-, and potentially DUF2169-containing proteins are required for loading a specific effector onto the cognate VgrG for delivery (Alcoforado Diniz and Coulthurst, 2015; Liang et al., 2015; Unterweger et al., 2015, 2016; Whitney et al., 2015; Bondage et al., 2016; Cianfanelli et al., 2016a). The DUF4123-containing protein functioning as a chaperone/adaptor was identified in the two studies in *Vibrio cholerae* with biochemical evidence showing its interaction with cognate VgrG (VgrG-1) and T6SS effector (TseL) by co-immunoprecipitation and bacterial two-hybrid analysis as well as genetic evidence for its requirement in mediating TseL secretion and TseL-mediated antibacterial activity (Liang et al., 2015; Unterweger et al., 2015). Together with its similar characteristics (i.e., low molecular weight protein with low isoelectric point, pI ~5) with chaperones in phages and the type III secretion system (T3SS), the DUF4123-containing protein was named the T6SS effector chaperone (TEC) (Liang et al., 2015). Its chaperone feature is consistent with previous observation of a DUF4123-containing protein, Atu4349 in stabilizing the Tde1 effector in *Agrobacterium tumefaciens* and forming a complex with Tde1 co-purified from *Escherichia coli* (Ma et al., 2014). However, because of its requirement for TseL binding to the VgrG-1 spike with no detectable effect on TseL stability, the DUF4123-containing protein was also named T6SS adaptor protein 1 (Tap-1) by considering its function as an adaptor for loading TseL to the VgrG-1 spike (Unterweger et al., 2015). Although Tap-1 can interact with VgrG-1 in the absence of TseL in *V. cholerae* (Unterweger et al., 2015), the *A. tumefaciens* Tap-1/TEC ortholog and Tde1 each cannot interact with its cognate VgrG (VgrG1) in the absence of each other, so the formation of this adaptor/chaperone-effector complex occurs before loading onto the VgrG spike (Bondage et al., 2016). The DUF1795-containing protein, named effector-associated gene (Eag), can specifically interact with the PAAR domain of the cognate effectors, which are stabilized by specific Eag proteins in *Serratia marcescens* and *Pseudomonas aeruginosa* (Alcoforado Diniz and Coulthurst, 2015; Whitney et al., 2015; Cianfanelli et al., 2016a). Furthermore, EagR1 and EagR2 each interact with the cognate Rhs effector with specificity (i.e., EagR1 binds to Rhs1 but not Rhs2 and vice versa) (Cianfanelli et al., 2016a). In the absence of Rhs2, EagR2 can no longer load onto its cognate VgrG spike (VirG2) (Cianfanelli et al., 2016a). These data strongly suggest that Eag functions as a chaperone perhaps with specificity for PAAR-containing effectors. Another putative chaperone family protein is the DUF2169-containing protein, which was first identified in *A. tumefaciens* Atu3641 for its role in stabilizing the PAAR-like DUF4150-containing Tde2 effector for Tde2-dependent antibacterial activity (Bondage et al., 2016). The widespread genetic linkage between DUF2169- and DUF4150/PAAR-containing genes in several T6SS^+^ proteobacterial genomes also implies a specific interaction between the two domains DUF2169 and DUF4150/PAAR. Taken together, Tap-1/TEC, Eag, and perhaps DUF2169-containing protein mainly function for loading specific effectors onto the cognate VgrG spike for secretion, but different adaptors/chaperones may have subtle differences in their modes of action, which awaits further structural and biochemical studies to clarify.

T6SS effectors with diverse biochemical activities have been identified. The major functions include the membrane-, cell wall-, or nucleic acid-targeting antibacterial effectors and several eukaryote-targeting effectors with a variety of enzymatic activities (Russell et al., 2014a; Alcoforado Diniz et al., 2015). In addition to the effectors functioning inside target cells, a few recent examples also suggested that T6SS effectors may function extracellularly rather than inside target cells. These identified extracellular effectors mainly function to bind or facilitate metal ions for their uptake (Wang et al., 2015; Lin et al., 2017; Si et al., 2017).

T6SS is widespread in Gram-negative bacteria, which include free-living bacteria and pathogens/symbionts of plants or animals. However, the number of identified effectors remains limited, in part because the annotation based on the primary genomic sequence information is often insufficient to properly annotate the function of effector genes. Nevertheless, almost all identified effectors are encoded with genetic linkage to the *vgrG* or *hcp* locus in the T6SS main gene cluster(s) or orphan *vgrG/hcp* island (De Maayer et al., 2011; Ma et al., 2014; Shyntum et al., 2014; Liang et al., 2015; Salomon et al., 2015; Abby et al., 2016). Various approaches have been successfully used to identify T6SS effectors. In this review, we summarize past and current methods as well as proposed potential strategies for identifying T6SS effectors. We hope such information can facilitate the discovery of novel T6SS effectors and elucidate their biochemical activities and biological functions.

## Past and current methods for identifying T6SS effectors

With the current knowledge of biochemical characteristics and transport mechanisms of T6SS effectors, T6SS effectors can be identified via both discovery-driven and knowledge/hypothesis-based methodologies. The methods used for identifying T6SS effectors are described as follows and summarized in Table 1, which also lists the pros and cons of these methods.

**Table 1:** Summary of current and potential methods for discovery of T6SS effectors.

**Method**	**Advantages**	**Limitation / Disadvantages**
**CURRENT METHOD**		
Bioinformatics analysis	Fast and robust in predicting putative effector candidates without experimental work	Prior knowledge or hypothesis is required
Genetic analysis of T6SS-associated genes	Easier to conduct without large-scale omics analysis or library construction and screening	Can only reveal effectors in the T6SS gene cluster or *hcp/vgrG* island
Proteomics-based method	Revealing effectors without known features	Limited to identifying proteins with significant secretion *in vitro* or a known chaperone for stability
Mutant library screening	Revealing effectors without known features; linking the gene to certain phenotypes directly	May identify genes other than T6SS effectors but with the same phenotypes; requires a testable phenotype
**POTENTIAL METHOD**		
Expression library	Revealing effectors without known features	Limited to identifying genes with cytosolic toxicity or screenable phenotype; may identify genes other than T6SS effectors
Protein–protein interactions	Revealing effectors interacting with known T6SS components	Limited to effectors with direct or tight interactions with bait protein; toxic protein may be harmful for the bacterial/yeast two-hybrid host

### Bioinformatics analysis

Some VgrG and Hcp proteins belong to specialized effectors by bearing a C-terminal effector domain (Pukatzki et al., 2009; Jamet and Nassif, 2015; Cianfanelli et al., 2016b; Ma et al., 2017a). Thus, VgrG or Hcp proteins with an additional C-terminal extension region are promising effector candidates with dual structural and biological roles. VgrG proteins can be classified into two categories: the canonical VgrG with gp27-gp5 domains as a sole structure function and the evolved VgrG with a C-terminal extended region conferring an additional domain(s) (Pukatzki et al., 2007). Therefore, several evolved VgrG proteins are identified, such as *V. cholerae* VgrG-1 containing an actin-crosslinking domain for a role in pathogenesis (Pukatzki et al., 2007) and the C-terminus of *V. cholerae* VgrG-3 with peptidoglycan degradation capability functioning as an antibacterial effector (Brooks et al., 2013; Dong et al., 2013). Besides bearing effector functions in the C-terminal extension of these evolved VgrG proteins, in entero-aggregative *E. coli* (EAEC), the VgrG1 harbors a C-terminal extension carrying DUF2345 and transthyretin (TTR) domains that are responsible for Tle1 effector binding (Flaugnatti et al., 2016). Co-immunoprecipitation (co-IP) and bacterial two-hybrid experiments suggested that the C-terminal extension is necessary and sufficient for interacting with the Tle1 effector, with the TTR domain involved in direct interaction with Tle1, whereas DUF2345 is required to stabilize the VgrG1–Tle1 interaction. In contrast to the widespread presence of evolved VgrG proteins harboring putative effector domains or an effector-binding domain in β-Proteobacteria and γ-Proteobacteria (Pukatzki et al., 2007; Boyer et al., 2009), Hcp proteins with a C-terminal extension have been found only in Enterobacteriaceae (Ma et al., 2017a). Although the C-terminal extension of some Hcp proteins was predicted to be a toxic domain, found in *Salmonella* in 2009 (Blondel et al., 2009), several Hcp proteins with a diverse C-terminal toxic domain (Hcp-ET) including Hcp-ET1 with DNase activity were recently characterized to exhibit antibacterial activity in Shiga toxin-producing *E. coli* (Ma et al., 2017a).

Besides searching for evolved VgrG or Hcp-ET, the conserved domains residing in known effectors can also be used as a query to search for potential effectors with available genome sequences. Russell et al. identified an amidase superfamily of T6SS effectors by searching for amidase catalytic cysteine and histidine residues combined with other features of T6SS effectors in their genome (Russell et al., 2012). With similar approaches, T6SS phospholipase effectors have been identified by the existence of a conserved motif, GXSXG, HXKXXXXD (Russell et al., 2013), or GXSXG (Flaugnatti et al., 2016). T6SS peptidoglycan glycoside hydrolase effectors were also identified by searching for the lysozyme-like fold (Whitney et al., 2013). Although they were not entirely identified by bioinformatics search, several nuclease effectors with predicted or verified conserved domains, such as the HXXD catalytic site motif of *A. tumefaciens* Tde (Zhang et al., 2012; Ma et al., 2014), HNH endonuclease domain of *S. marcescens* Rhs2 (Alcoforado Diniz and Coulthurst, 2015) and *Dickeya dadantii* RhsB (Koskiniemi et al., 2013), can be used to identify new T6SS effectors in any T6SS^+^ bacterial genome via bioinformatics tools.

T6SS effectors also possess motifs or domains related to functions involved in translocation or other structural functions rather than biochemical or biological activity. In 2012, Zhang et al. proposed that a bacterial toxin could be divided into three parts: an N-terminal trafficking associated region, a central region, and a C-terminal toxic region. Rearrangement hotspot (Rhs)/YD repeats are common features of a central region in a toxin protein transported by diverse mechanisms. However, the PAAR domain is mostly located at the N-terminus of a T6SS effector, although some of these domains are followed by an N-terminal extension region (Zhang et al., 2012; Shneider et al., 2013). Of note, some classes of PAAR-containing proteins also harbor an Rhs region and a TTR domain located at the C-terminal extension region (Zhang et al., 2012; Shneider et al., 2013). Because the TTR domain of VgrG1 protein in EAEC is necessary and sufficient for Tle1 effector binding (Flaugnatti et al., 2016), the TTR domain of some PAAR-containing proteins may function as a carrier for effectors as suggested (Shneider et al., 2013). Indeed, several T6SS effectors were identified to have these characteristics. *P. aeruginosa* Tse5/RhsP1 and RhsP2 are typical Rhs effectors (Hachani et al., 2014; Whitney et al., 2014), whereas *S. marcescens* Rhs1 and Rhs2 (Alcoforado Diniz and Coulthurst, 2015), *D. dadantii* RhsA and RhsB (Koskiniemi et al., 2013), and Rhs-CT in Shiga toxin-producing *E. coli* (Ma et al., 2017b) harbor both N-terminal PAAR and central Rhs domains. Many T6SS effectors are featured with an N-terminal PAAR domain followed by a C-terminal effector domain. The examples are *P. aeruginosa* Tse6/PA0093 carrying the typical PAAR (Hachani et al., 2014; Whitney et al., 2014); *A. tumefaciens* Tde2 (Ma et al., 2014) and *P. aeruginosa* PA0099 (Hachani et al., 2014) with the N-terminal PAAR-like domain DUF4150; and *Francisella novicida* IglG, an effector of non-canonical T6SS, containing another PAAR-like domain DUF4280 (Rigard et al., 2016). In addition to the Rhs/YD repeat and PAAR, some T6SS effectors possess the motif named marker for type six effectors (MIX), which was first found in *Vibrio parahaemolyticus* by a proteomics approach (Salomon et al., 2014). Further bioinformatics analysis revealed that this MIX motif is present in some proteins encoded within T6SS gene clusters or the *hcp*/*vgrG* island in T6SS^+^ Proteobacteria. Moreover, these MIX effectors may be horizontally transferred among marine bacteria (Salomon et al., 2015; Salomon, 2016). However, whether the MIX motif is important for the effector function or translocation remains to be tested. With these findings, PAAR- and Rhs-containing effectors were predicted to be widespread in Proteobacterial genomes, indicating that the use of bioinformatics tools to identify the presence of the Rhs/YD repeat, PAAR, TTR, and MIX motifs as a preliminary screen for identifying T6SS effectors or their interacting proteins is a promising strategy, although these motifs are not exclusively related to T6SS.

Because of an essential role required in mediating the transport of specific cargo effectors, the conserved domains of the known T6SS adaptors/chaperones including DUF4123 of Tap-1/TEC (Liang et al., 2015; Unterweger et al., 2015; Bondage et al., 2016), DUF1795 of Eag (Whitney et al., 2015; Cianfanelli et al., 2016a), and DUF2169 (Bondage et al., 2016) can be used to identify the cognate effector gene due to their close genetic linkage. Indeed, the conservation of these chaperones/adaptors with genetic linkage to the cognate effector genes has led to identification of a new T6SS effector, such as the TseC effector mediated by TEC in *A. hydrophila*, by searching for DUF4123 in the genome (Liang et al., 2015) and several putative effectors that are genetically linked to DUF4123 and DUF2169 found in many Proteobacterial genomes (Liang et al., 2015; Bondage et al., 2016). Thus, by identifying the conserved domains of these chaperone/adaptor genes, one can identify the putative effector genes and perform functional assays to confirm their effector functions and relationship with the cognate chaperone/adaptor and VgrG spike.

In addition to the above-mentioned methods specifically for T6SS effector prediction, some online bioinformatics tools or databases provide a suggestion for potential effectors, such as an effector protein predictor, secretEPDB (An et al., 2016, 2017); a T6SS database; SecReT6 (Li et al., 2015); and T346hunter, which can annotate homologs of type III, IV and VI secretion systems automatically (Martínez-García et al., 2015). For researchers working on *Burkholderia* spp., DBSecSys is an online database for the *Burkholderia mallei* and *Burkholderia pseudomallei* secretion system (Memišević et al., 2014, 2016). The Pfam database also provides a resource to identify potential effectors with conserved effector domains (Finn et al., 2014). In conclusion, the bioinformatics-based approach to identify new T6SS effectors is a convenient and powerful tool for study of organisms with a sequenced genome. However, bioinformatics is limited to our current knowledge of biochemical features and transport mechanisms of T6SS effectors.

### Genetic analysis of T6SS-associated genes

Some open reading frames in the T6SS main cluster encoding a hypothetical protein with unknown function could be candidate genes for T6SS effector or immunity proteins; combining deletion of these genes and phenotypic analysis such as interbacterial competition assay or virulence assay may lead to effector discovery. Hcp secretion is considered a hallmark of a functional T6SS and therefore can be used to determine the effects of the deletion mutant in T6SS assembly. In *S. marcescens*, two genes with unknown function in the T6SS gene cluster were found to be T6SS effectors, *ssp1* and *ssp2*, via bacterial competition assay and protein–protein interaction studies (English et al., 2012). The other case is the Tae and Tde1 effectors in *A. tumefaciens* C58, in which both Tae and Tde1 are secreted proteins, and their absence does not affect Hcp secretion (Lin et al., 2013; Ma et al., 2014). Follow-up experiments further demonstrated their functions as bacterial toxins and that their cognate immunity proteins are encoded from their adjacent genes (Ma et al., 2014). Some effectors are not encoded in the T6SS main cluster but are found in the *hcp/vgrG* island located outside the T6SS main cluster (Abby et al., 2016). Such effector genes include *tse5/rhsP1* and *rhsP2* in *P. aeruginosa*, found near *vgrG4* and *vgrG14*, respectively (Hachani et al., 2014; Whitney et al., 2014); *tde2* in *A. tumefaciens* C58, located in the *vgrG2* operon (Ma et al., 2014); and some effectors found located in the *vgrG*/*hcp* island in *Pantoea* and *Erwinia* species (De Maayer et al., 2011). Therefore, hypothetical proteins encoded in the T6SS gene cluster or orphan *vgrG/hcp* island can be analyzed genetically by virulence assay, antibacterial competition assay and/or protein secretion. Together with sequence- and structure-based search engines, such as NCBI blast searches (Ncbi Resource Coordinators, 2017) and Phyre2 (Kelley et al., 2015), one can identify T6SS effectors without large-scale omics approaches or library construction and screening.

### Proteomics-based method

Unlike the bioinformatics-based method depending on empirical known characteristics of T6SS effectors, proteomics is a discovery-driven approach that can identify T6SS effectors without prior knowledge. A simple experimental design is to identify T6SS effector candidates via a comparative proteomics analysis between a wild-type strain and a T6SS secretion-deficient mutant. The *P. aeruginosa* H1-T6SS effectors Tse1, Tse2, and Tse3 were found by comparing the secretome between the hyper-secreted *pppA* deletion mutant and *clpV1* secretion-deficient mutant (Hood et al., 2010). Similarly, a gel-free secretome analysis comparing the wild-type strain and *clpV* mutant of *V. cholerae* revealed a TseH effector possessing an amidase domain and two YD repeats (Altindis et al., 2015). The mutant of *vasH*, encoding a transcriptional regulator of T6SS (Kitaoka et al., 2011), was used to identify the new effectors VasX and VgrG1 in *V. cholerae* and *A. hydrophila*, respectively (Suarez et al., 2010; Miyata et al., 2011). Both of these effectors were confirmed by a virulence assay with amoeba or HeLa cells as models. The use of the *tssC* mutant as a T6SS secretion-deficient mutant to analyze the comparative secretome also led to the discovery of Tae2 in *B. thailendensis*, Bte1 and Bte2 in *Burkholderia fragilis* and Fte2 in *Flavobacterium johnsoniae* (Russell et al., 2012, 2014b; Wexler et al., 2016). Comparison of the secretomes of the *S. marcescens clpV* mutant and the wild type revealed four new effectors: Ssp3, Ssp4, Ssp5, and Ssp6 (Fritsch et al., 2013). The effector function of Ssp4 was confirmed by interbacterial competition assay; the toxic effects of others were determined by examining the survival of effector-expressing *E. coli* cells. Comparison of the secretomes of the T6SS cluster-deletion mutant and the wild type of enterohemorragic *E. coli* (EHEC) revealed a magnesium (Mn)-containing catalase, KatN, as a T6SS effector (Wan et al., 2017). This study demonstrated that KatN is secreted in a T6SS-dependent secretion to the extracellular milieu *in vitro* and into the host cell cytosol after EHEC is phagocytized by macrophages. Interestingly, *katN* is not located in the T6SS cluster or the *hcp/vgrG* island. Thus, determining the molecular mechanisms underlying how KatN is transported via T6SS through VgrG or Hcp as a carrier or via another yet-to-be discovered mechanism would be of interest. In addition, secretome analysis of each specific *vgrG* mutant in *S. marcescens* was used to identify a VgrG-dependent effector, which led to the discovery of an Slp effector in *S. marcescens* with VgrG2-dependent secretion (Cianfanelli et al., 2016a).

The characteristics of a chaperone that stabilizes the cognate effector can also be used to identify new T6SS effectors. This idea led to the identification of Tse4 in *P. aeruginosa* (Whitney et al., 2014). Because Hcp1 in *P. aeruginosa* has the chaperone activity that stabilizes effectors (Silverman et al., 2013), comparing the intracellular proteome between the wild type and Δ*hcp1* was used as a unique method to identify candidate effectors with lower accumulation in Δ*hcp1* (Whitney et al., 2014). This gave us a hint that the same principle can be used to identify the effector requiring a cognate chaperone for stability by performing comparative cellular proteome analysis between the wild type and the mutant deficient in a specific chaperone, such as a DUF4123-, DUF2169-, or DUF1795-containing protein.

The proteomics-based method is a powerful tool to identify potential effectors in a hypothesis-driven or knowledge-independent manner. Thus, this approach may identify novel effectors with conserved motifs such as MIX motif-containing effectors found in *V. parahaemolyticus* (Salomon et al., 2014). However, since not all effectors can be secreted *in vitro* in significant amounts or require a chaperone for stability, current proteomics-based methods are limited to identify effectors with these criteria.

### Mutant library screening

In addition to proteomics approaches, mutant library screening is another powerful tool to identify T6SS effectors without known features yet is often a phenotype-dependent method. Of note, the very first T6SS reported in *V. cholerae* was identified by this approach, although discovery of a secretion system was not intentional. The key to identifying the T6SS involved in *V. chalerae* virulence is the use of a novel bacterial virulence assay with the eukaryotic amoebae organism *Dictyostelium discoiseum* as a host model system, which uptakes bacterial cells by phagocytosis, mimicking the behavior of macrophages (Steinert and Heuner, 2005; Pukatzki et al., 2006). The transposon insertion mutants, *vasH, vasA* (*tssF*), and *vasK* (*tssM*), were found to lose the anti-amoebae activity and later found deficient in secretion of a previously known secreted protein, Hcp (Williams et al., 1996; Pukatzki et al., 2006). Thus, a robust phenotype screening system is required for identifying T6SS effectors by mutant library screening. In *Burkholderia cenocepacia*, the T6SS effector TecA was found by selecting the transposon insertion mutants that cannot disrupt the actin of macrophages, and TecA was further demonstrated to function as a Rho-GTPase deaminase (Aubert et al., 2016). Because the immunity protein is essential for resistance against wild-type siblings with antibacterial activity but not T6SS-deficient siblings, Dong et al. used transposon insertion-site sequencing (Tn-seq) to identify the immunity gene and associated effector gene in a transposon mutant library screening (Dong et al., 2013). By this approach, they identified a new T6SS effector with lipase domain, TseL, and two known effectors: VgrG3, with peptidoglycan degradation activity, and VasX, which may interact with phospholipids. Because this study focused on identifying mutants that are absent in T6SS^+^ but present in T6SS^−^ *V. cholerae* strains, the mutants that are lethal in both T6SS^+^ and T6SS^−^ strains could not be selected. As a result, all three effectors identified are cell wall– or membrane-targeting effectors because the effectors only exhibit the function when delivered to the cell wall or membrane of target cells; thus, the respective immunity mutants remain viable for selection. For the effectors with cytosolic targets, one may identify the cognate immunity gene by directly sequencing the saturating mutant library because of the lethality of the mutants. Tn-seq technology was also used to identify a *V. cholerae* transposon insertion *tsiV3* mutant that cannot survive in the infant rabbit gut, which suggests a role for T6SS in antagonistic interbacterial interactions during infection (Fu et al., 2013). The *tsiV3* mutant of this *V. cholera* strain C6706 is viable when grown in regular growth medium because T6SS is not expressed during *in vitro* growth but is induced *in vivo* for bacterial colonization in the animal host.

Mutant library screening has been successfully used to identify T6SS effectors or cognate immunity, but mutant strains with impaired competitive growth or virulence phenotypes could be caused by mutation in genes involved in T6SS machine assembly or even other virulence-associated factors. In *B. pseudomallei*, transposon insertion mutant library screening revealed the T3SS as the main system related to host virulence, whereas T6SS-1 is important for the virulence in the Madagascar hissing cockroach model, and T6SS-5 may be associated with fitness in mice (Fisher et al., 2012; Gutierrez et al., 2015). Library screening approaches also identified T6SS associated with other phenotypes. For example, a transposon insertion in *clpV* highly attenuated the secretion of iron chelator pyoverdine from *Pseudomonas taiwanensis* with reduced antagonism ability (Chen et al., 2016). Transposon insertion mutations in *vgrG* or *hcp* operons in *Proteus mirabilis* altered swarming behavior (Alteri et al., 2013). However, whether the impact on pyoverdine secretion or swarming ability are the direct phenotype or are secondary effects due to the absence of T6SS await further validation.

## Potential methods for identifying T6SS effectors

In addition to the above-mentioned methods that have been successfully used to identify T6SS effectors directly or indirectly, we discuss two potential approaches that may help to identify T6SS effectors.

### Expression library

Most of the T6SS effectors identified to date are usually toxic to eukaryotic hosts or competitor bacteria. Thus, we can take advantage of such characteristics to identify effector genes by screening a genome-wide expression library in yeast (for eukaryotic toxin) or *E. coli* cells (for bacterial toxins). Indeed, since the 2000s, yeast has been used as a model to find bacterial effectors of other secretion systems (Siggers and Lesser, 2008). The key is to use the tightly regulated inducible promoter to express the genes of interests and screen the construct with growth inhibition or arrest under inducible condition. This kind of approach has been used to identify T3SS effectors in *Shigella* sp., *P. aeruginosa* and *Xanthomonas campestris* pv. *vesicatoria*. In *Shigella* sp., a T3SS effector, IpaJ, encoded from *Shigella* virulence plasmids, was found to be toxic and inhibit growth when expressed in yeast cells and later confirmed to have T3SS-dependent secretion (Slagowski et al., 2008). A genome-wide expression library constructed from *P. aeruginosa* was transformed into yeast cells, and the expressed proteins leading to yeast cell death or growth inhibition after induction were selected for further virulence assay by infecting *Caenorhabditis elegans* and macrophages with wild-type *P. aeruginosa* or the respective deletion mutants. Such screening indeed identified several virulence factors of *P. aeruginosa*, but the delivery mechanism of these proteins has yet to be determined (Zrieq et al., 2015). In *X. campestris* pv. *vesicatoria*, this approach was used to determine the biological function of already identified T3SS effectors (Salomon et al., 2010). In addition to performing the screening experiments under normal growth conditions, various stress conditions can be used with transformants to identify the effectors targeting the conserved pathway activated only under stress (Slagowski et al., 2008; Salomon et al., 2010).

Toxin effector screening has been also performed in prokaryotic cells. By transforming a DNA library from 388 microbial genomes into *E. coli* and sequencing all surviving transformants, the gene (toxin) that could survive only when its adjacent gene is present on the same clone (antitoxin) was identified (Sberro et al., 2013). Hence, Sorek's group identified both known (238 pairs) and newly identified (123 pairs) toxin/antitoxin pairs. Thus, in view of the toxicity of T6SS effectors and co-existence relationship of the toxin-immunity pair, one can use the inducible expression library and toxin-immunity pair for survival screening to identify T6SS effectors. However, if the effector only exerts its function on the cell membrane/surface or extracellularly, its effector function cannot be uncovered because it may require the T6SS machine for delivery to the right compartments. Therefore, expression library screening may only identify effectors with a cytoplasmic target unless a signal peptide is fused to the expressed proteins for translocation across the inner membrane. Also, the expression library is not specifically designed for T6SS effector identification. Another may also identify genes unrelated to T6SS. Therefore, after identification of putative effector candidates, further elucidation of the delivery mechanism is required to conclude the genes encoding T6SS effectors. One limitation in the expression library is that effectors with subtle effects or other physiological functions not related to cell growth may be overlooked.

### Protein–protein interaction

As described previously, several T6SS cargo effectors are loaded to the VgrG spike via non-covalent binding with chaperone or adaptor proteins such as Eag or Tap-1/TEC (Pukatzki et al., 2009; Liang et al., 2015; Unterweger et al., 2015, 2016; Whitney et al., 2015; Bondage et al., 2016; Cianfanelli et al., 2016b). Others such as Tse2 in *P. aeruginosa* and Tae4 in *Salmonella typhimurium* are secreted by binding to the lumen of the Hcp hexamer (Silverman et al., 2013; Sana et al., 2016). Therefore, the T6SS effectors were found to be directly or indirectly associated with Hcp, VgrG, PAAR, Tap-1/TEC, or Eag in various bacteria (Shneider et al., 2013; Silverman et al., 2013; Hachani et al., 2014; Liang et al., 2015; Unterweger et al., 2015; Whitney et al., 2015; Bondage et al., 2016; Cianfanelli et al., 2016a; Rigard et al., 2016). Because the genes encoding these conserved T6SS components functioning as carriers or chaperones/adaptors for T6SS effectors are easier to be predicted than most effector genes in a sequenced genome, these proteins can be first identified and used as a bait in well-established protein–protein interaction platforms such as bacterial two-hybrid (Battesti and Bouveret, 2012), yeast two-hybrid (Mehla et al., 2015), or co-IP/pull-down assay (Brymora et al., 2004; Kaboord and Perr, 2008) to identify potential effectors. This idea was indeed proposed by Silverman et al., that interaction with the Hcp chaperone may be a novel method to identify new T6SS effectors (Silverman et al., 2013). One disadvantage of this method is that it can identify only effectors that directly or tightly bind with adaptors/chaperones or the T6SS machine. Because expression of the effector toxin may be harmful for the host used in bacterial or yeast two-hybrid assays, some key effector toxins may be missed by such screening.

## Biochemical and biological functions of known T6SS effectors

Numerous T6SS effectors have been identified with experimentally proven or predicted biochemical activities. In general, the effectors can be classified by their target cells as eukaryotic hosts or bacterial competitors, although the mode of action or biochemical function of effectors can be distinct or shared between eukaryotic or prokaryotic effectors (Table 2). So far, the major biological functions of these effectors are to increase the competitive growth advantages of environmental or host-associated bacteria or virulence of the bacterial pathogen (Kapitein and Mogk, 2013; Durand et al., 2014; Russell et al., 2014a; Alcoforado Diniz et al., 2015; Hachani et al., 2016). In addition to functioning as a contact-dependent weapon against the host or bacterial competitor, some T6SSs may secrete effectors for scavenging cofactors to survive in oxidative stress and/or facilitate metal ion acquisition (Chen et al., 2016; Lin et al., 2017; Si et al., 2017; Wan et al., 2017). These findings suggest diversified T6SS functions for bacterial survival and fitness in its ecological niches.

**Table 2:** Known T6SS effectors with defined biochemical activities.

**Effector**	**Organism**	**Biochemical activity**	**References**
**EUKARYOTIC TARGET**			
VgrG1	*A. hydrophila*	ADP-ribosyltransferase	Suarez et al., 2010
TecA	*B. cenocepacia*	Deaminating Rho GTPase	Aubert et al., 2016
VgrG-5	*B. thailendensis*	Membrane fusion activity	Schwarz et al., 2014
EvpP	*E. tarda*	Inhibition of NLRP3 inflammasome	Yang et al., 2015; Chen et al., 2017
VgrG-1	*V. cholerae*	Actin-crosslinking	Pukatzki et al., 2007; Ma and Mekalanos, 2010
VgrG2b	*P. aeruginosa*	Interacting with microtubule	Sana et al., 2015
KatN	Enterohemorragic *E. coli*	Mn-containing catalase	Wan et al., 2017
**DUAL TARGET**			
PldA/Tle5, PldB	*P. aeruginosa*	Phospholipase, activation of Akt signaling pathway	Russell et al., 2013; Jiang et al., 2014
VasX	*V. cholerae*	Membrane-targeting activity	Dong et al., 2013; Miyata et al., 2013
TseL/Tle2	*V. cholerae*	Phospholipase	Dong et al., 2013; Russell et al., 2013
**BACTERIAL TARGET—LIPASE ACTIVITY**			
Tle1	*Burkholderia thailandensis*	Phospholipase	Russell et al., 2013
Tle1	Entero-aggregative *E. coli*	Phospholipase	Flaugnatti et al., 2016
**BACTERIAL TARGET—CELL WALL DEGRADATION ENZYME**			
Tse1	*P. aeruginosa*	Amidase	Hood et al., 2010; Russell et al., 2011
Tse3	*P. aeruginosa*	Muramidase	Hood et al., 2010; Russell et al., 2011
TseH	*V. cholerae*	Cell-wall degradation hydrolase	Altindis et al., 2015
VgrG-3	*V. cholerae*	Peptidoglycan degradation	Pukatzki et al., 2006; Brooks et al., 2013
Tge2	*P. aeruginosa*	Glycoside hydrolase	Whitney et al., 2013
**BACTERIAL TARGET—DNASE**			
Tde1, Tde2	*A. tumefaciens*	DNase	Ma et al., 2014
RhsA, RhsB	*D. dadantii*	DNase	Koskiniemi et al., 2013
Hcp-ET1	Shiga toxin-producing *E. coli*	DNase	Ma et al., 2017a
Rhs2	*S. marcescens*	DNase	Alcoforado Diniz and Coulthurst, 2015
**BACTERIAL TARGET—OTHER BIOCHEMICAL ACTIVITY**			
Tse2	*P. aeruginosa*	NAD-dependent toxicity	Hood et al., 2010; Robb et al., 2016
Tse6	*P. aeruginosa*	NAD(P)+ glycohydrolase	Whitney et al., 2014, 2015
**EXTRACELLULAR**			
TseM	*B. thailendensis*	Mn^2+^-binding protein	Si et al., 2017
YezP	*Y. pseudotuberculosis*	Zinc-binding protein	Wang et al., 2015
TseF	*P. aeruginosa*	Bind OMV for iron acquisition	Lin et al., 2017

The biological and biochemical functions of antihost T6SS effectors are quite diverse. Some of the virulence-associated T6SS effectors can interact with the cytoskeleton of the host cells directly. *V. cholerae* VgrG-1 can cause actin crosslinking, which is associated with the intestinal inflammation (Pukatzki et al., 2007; Ma and Mekalanos, 2010). *P. aeruginosa* VgrG2b interacts with microtubules causing the successful invasion of epithelial cells (Sana et al., 2015). VgrG1 in *A. hydrophila* has ADP-ribosyltransferase activity and can disrupt the cytoskeleton of HeLa cells (Suarez et al., 2010). Others can interact with the plasma membrane of host cells. *B. thailandensis* VgrG-5 has membrane fusion activity and is required for the multinucleated giant cell formation to spread between cells (Schwarz et al., 2014). Besides targeting the host cytoskeleton or membrane, an Mn-containing catalase, KatN, was shown to be translocated from the enterohemorrhagic *E. coli* (EHEC) into host cells in a T6SS-dependent manner and reduce the reactive oxygen species concentration in host macrophages in a T6SS-dependent manner (Wan et al., 2017). Although T6SS appears to be important for EHEC virulence, as demonstrated in mouse model, no virulence phenotype could be observed in the *katN* mutant. In *B. cenocepacia*, TecA deaminates Rho GTPase in host cells and triggers the inflammation reaction and actin disruption (Aubert et al., 2016). Although infection of the *tecA* mutant will not trigger inflammation, it can kill mice, as compared with the survival of all mice infected with wild-type *B. cenocepacia*. The authors proposed that TecA may limit the bacterial cell number in the host and cause the bacteria to evolve toward a mutual relationship with the host. In contrast, the fish pathogen *Edwardsiella tarda* produces a unique T6SS effector EvpP, which does not harbor any known functional domain, to suppress inflammasome activation and promote colonization in the host (Chen et al., 2017). Thus, bacterial pathogens seem to evolve different T6SS effectors in modulating the host immunity for survival and fitness, but much more remains to be learned about their host targets and mode of actions (Yu and Lai, 2017).

The antibacterial T6SS effectors identified so far can be divided into membrane-, cell wall-, and nucleic acid-targeting effectors as well as other biological functions (Durand et al., 2014; Russell et al., 2014a; Alcoforado Diniz et al., 2015). Membrane-targeting effectors include Tle phospholipase superfamily such as *P. aeruginosa* PldA/Tle5, *V. cholerae* Tle2/TseL, and *Burkholderia thailandensis* Tle1 (Russell et al., 2013) and entero-aggregative *E. coli* Tle1 (Flaugnatti et al., 2016). VgrG3 and TseH in *V. cholerae* (Brooks et al., 2013; Altindis et al., 2015) and Tse1 amidase and Tse3 muramidase (Russell et al., 2011) in *P. aeruginosa* belong to cell wall-targeting effectors. *S. marcescens* Rhs2 (Alcoforado Diniz and Coulthurst, 2015), *D. dadantii* RhsA and RhsB (Koskiniemi et al., 2013), *A. tumefaciens* Tde1 and Tde2 (Ma et al., 2014), and Shiga toxin-producing *E. coli* Hcp-ET1 (Ma et al., 2017a) can hydrolyze DNA. Some effectors have other toxic effects in bacterial cytoplasm; for example, *P. aeruginosa* Tse6 is an NAD(P)+ glycohydrolase toxin inducing bacteriostasis by depleting cellular NAD(P)+ levels (Whitney et al., 2015) and Tse2 is likely an NAD-dependent enzyme from the structure information (Robb et al., 2016).

Many T6SS effectors can be secreted into the extracellular milieu during *in vitro* culture, but these antihost or antibacterial toxin effectors exhibit their effector functions inside the target cells. A few reports recently provided compelling biochemical evidence for some T6SS effectors functioning extracellularly rather than in target cells. These effectors so far identified mainly function to bind or facilitate metal ions for their uptake. *Yersinia pseudotuberculosis* T6SS-4 encodes a unique zinc-binding effector protein, YezP, that is involved in environmental stress resistance in the host body (Wang et al., 2015). The double mutant lacking this effector and classical zinc transporter exhibits almost no virulence toward mice. Similar to *Y. pseudotuberculosis*, TseM secreted by T6SS-4 of *Burkholderia thailandensis* functions as an Mn^2+^-binding protein in interacting with the Mn^2+^-specific TonB-dependent outer-membrane protein MnoT to activate transport for resistance to oxidative stress (Si et al., 2017). Deletion of *tseM* caused reduced virulence toward *Galleria mellonella* wax moth larvae. Although not functioning as a metal-binding protein, TseF secreted by H3-T6SS of *P. aeruginosa* can bind to and recruit outer-membrane vesicles for iron (Fe) acquisition (Lin et al., 2017). By engaging the Fe(III)-pyochelin receptor FptA and the porin OprF, TseF can facilitate the delivery of outer-membrane vesicle-associated Fe for bacterial cells.

Although many effectors target only eukaryotic cells (i.e., *V. cholerae* VgrG-1 with an actin cross-linking domain) or prokaryotic cells (i.e., *P. aeruginosa* Tse1 amidase and Tse3 muramidase for targeting peptidoglycan), some effectors may have dual targets. PldA/Tle5 and PldB, phospholipase D effectors secreted from *P. aeruginosa*, can target both bacterial and eukaryotic cells (Jiang et al., 2014). Both PLD effectors can inhibit bacterial growth by increasing membrane permeability when translocated into the periplasm of bacterial recipient cells while activating phosphorylation of the protein kinase B (Akt) signaling pathway to lead to the internalization of the bacteria into the host cell. TseL/Tle2 in *V. cholerae* is phospholipase, which exhibits both T6SS-dependent antibacterial activity and a virulence role toward *D. discoideum* (Dong et al., 2013; Russell et al., 2013). In addition, *V. cholerae* VasX is a membrane targeting toxin that can interact with phosphoinositides important for virulence toward the eukaryotic amoebae *D. discoideum* and compromise the integrity of the inner membrane in bacterial target cells for antibacterial activity (Miyata et al., 2011, 2013; Dong et al., 2013). The *P. aeruginosa* Tse2 toxin naturally attacks bacterial cells for interbacterial competition but can also cause toxicity when ectopically expressed in eukaryotic cells (Robb et al., 2016). Thus, although current data show that the DNase effectors such as Tde and NAD(P)+ glycohydrolase effectors such as Tse6 are involved in antibacterial activity, whether they can also be translocated into eukaryotic host cells remains to be tested. In considering the role of T6SS in bacterial colonization inside hosts such as *A. tumefaciens* T6SS for competitive growth advantage *in planta* (Ma et al., 2014), *V. cholera* T6SS for survival in the infant rabbit gut (Fu et al., 2013), and T6SS for survival of the bacterial symbionts Bacteroidetes in human guts (Wexler et al., 2016), such possibility could be explored.

## Perspectives

The T6SS is known to have important roles in bacterium–host interaction, bacterium–bacterium interaction and even other functions associated with bacterial physiology. Considering the expanded and diversifying functions of the T6SS discovered since its identification more than a decade ago, the system may have more functions, especially biological significance at polymicrobial ecological niches yet to be uncovered. The methodologies and biology of T6SS effectors we discuss in this review can be a foundation for future identification and studies of the T6SS and effectors.

## Author contributions

YL and EL discussed the review content and wrote the paper together.

### Conflict of interest statement

The authors declare that the research was conducted in the absence of any commercial or financial relationships that could be construed as a potential conflict of interest.
